# An Ocean-Colour Time Series for Use in Climate Studies: The Experience of the Ocean-Colour Climate Change Initiative (OC-CCI)

**DOI:** 10.3390/s19194285

**Published:** 2019-10-03

**Authors:** Shubha Sathyendranath, Robert J.W. Brewin, Carsten Brockmann, Vanda Brotas, Ben Calton, Andrei Chuprin, Paolo Cipollini, André B. Couto, James Dingle, Roland Doerffer, Craig Donlon, Mark Dowell, Alex Farman, Mike Grant, Steve Groom, Andrew Horseman, Thomas Jackson, Hajo Krasemann, Samantha Lavender, Victor Martinez-Vicente, Constant Mazeran, Frédéric Mélin, Timothy S. Moore, Dagmar Müller, Peter Regner, Shovonlal Roy, Chris J. Steele, François Steinmetz, John Swinton, Malcolm Taberner, Adam Thompson, André Valente, Marco Zühlke, Vittorio E. Brando, Hui Feng, Gene Feldman, Bryan A. Franz, Robert Frouin, Richard W. Gould, Stanford B. Hooker, Mati Kahru, Susanne Kratzer, B. Greg Mitchell, Frank E. Muller-Karger, Heidi M. Sosik, Kenneth J. Voss, Jeremy Werdell, Trevor Platt

**Affiliations:** 1 National Centre for Earth Observation, Plymouth Marine Laboratory, Prospect Place, Plymouth PL1 3DH, UK; robr@pml.ac.uk; 2 Brockmann Consult, Max-Planck-Straße 2, D-21502 Geesthacht, Germany; carsten.brockmann@brockmann-consult.de (C.B.); dagmar.mueller@brockmann-consult.de (D.M.); marco.zuehlke@xing.com (M.Z.); 3 Marine Environmental Sciences Centre, Faculdade de Ciências, Universidade de Lisboa, Campo Grande, 1749-016 Lisboa, Portugal; vbrotas@fc.ul.pt (V.B.); belocouto@gmail.com (A.B.C.); asfg.valente@gmail.com (A.V.); 4 PML Applications Ltd, Prospect Place, Plymouth PL1 3DH, UK; bac@pml.ac.uk; 5 Plymouth Marine Laboratory, Prospect Place, Plymouth PL1 3DH, UK; ach@pml.ac.uk (A.C.); jad@pml.ac.uk (J.D.); michael.grant@eumetsat.int (M.G.); sbg@pml.ac.uk (S.G.); a.horseman@computer.org (A.H.); thja@pml.ac.uk (T.J.); vmv@pml.ac.uk (V.M.-V.); chs@pml.ac.uk (C.J.S.); Malcolm.Taberner@external.eumetsat.int (M.T.); arthompson1990@gmail.com (A.T.); tplatt@dal.ca (T.P.); 6 Telespazio Vega UK for ESA Climate Office, European Space Agency/ECSAT, Harwell Campus OX11 0FD, UK; paolo.cipollini@esa.int; 7 Helmholtz-Zentrum Geesthacht, Zentrum für Material- und Küstenforschung GmbH, Max-Planck-Straße 1, D-21502 Geesthacht, Germany; roland.doerffer@hzg.de (R.D.); hajo.krasemann@hzg.de (H.K.); 8 European Space Agency/ESTEC, Keplerlaan 1, 2201 AZ Noordwijk, The Netherlands; craig.donlon@esa.int; 9 European Commission, Joint Research Centre (JRC), Via Enrico Fermi, 2749, I-21027 Ispra, Italy; mark.dowell@ec.europa.eu (M.D.); Frederic.MELIN@ec.europa.eu (F.M.); 10 Telespazio VEGA UK Ltd., 350 Capability Green, Luton, Bedfordshire LU1 3LU, UK; Alex.Farman@telespazio.com (A.F.); Sam.Lavender@telespazio.com (S.L.); john.swinton@telespazio.com (J.S.); 11 Solvo, 3 rue Saint-Antoine, 06600 Antibes, France; constant.mazeran@solvo.fr; 12 Ocean Process Analysis Laboratory, Morse Hall, University of New Hampshire, Durham, NH 03824, USA; tsmoore00@gmail.com (T.S.M.); hui.feng@unh.edu (H.F.); 13 European Space Agency, ESRIN, Via Galileo Galilei, Casella Postale 64, 00044 Frascati (Roma), Italy; pregner@iwind.it; 14 Department of Geography and Environmental Sciences, University of Reading, Whiteknights, Reading RG6 6DW, UK; shovonlal.roy@reading.ac.uk; 15 HYGEOS, 165 Avenue de Bretagne, 59000 Lille, France; fs@hygeos.com; 16 CNR-ISMAR, Via Fosso del Cavaliere, 100, 00133 Roma, Italy; 17 NASA Goddard Space Flight Center, Greenbelt, MD 20771, USA; gene.c.feldman@nasa.gov (G.F.); bryan.a.franz@nasa.gov (B.A.F.); stanford.b.hooker@nasa.gov (S.B.H.); jeremy.werdell@nasa.gov (J.W.); 18 Scripps Institution of Oceanography Mail Code 0218, University of California San Diego, La Jolla, CA 92039-0218, USA; rfrouin@ucsd.edu (R.F.); mkahru@ucsd.edu (M.K.); gmitchell@ucsd.edu (B.G.M.); 19 Naval Research Laboratory, Bldg. 1009, Code 7331, Stennis Space Center, MS 39529, USA; gould@nrlssc.navy.mil; 20 Department of Ecology, Environment and Plant Sciences, University of Stockholm, 106 91 Stockholm, Sweden; susanne.kratzer@su.se; 21 Institute for Marine Remote Sensing, College of Marine Science, University of South Florida, 140 7th Ave. South St, Petersburg, FL 33701, USA; carib@usf.edu; 22 Biology Department, MS 32, Woods Hole Oceanographic Institution, Woods Hole, MA 02543-1049, USA; hsosik@whoi.edu; 23 Department of Physics, University of Miami, James L. Knight Physics Building, 1320 Campo Sano Dr., Coral Gables, FL 33124, USA; voss@physics.miami.edu

**Keywords:** ocean colour, water-leaving radiance, remote-sensing reflectance, phytoplankton, chlorophyll-a, inherent optical properties, Climate Change Initiative, optical water classes, Essential Climate Variable, uncertainty characterisation

## Abstract

Ocean colour is recognised as an Essential Climate Variable (ECV) by the Global Climate Observing System (GCOS); and spectrally-resolved water-leaving radiances (or remote-sensing reflectances) in the visible domain, and chlorophyll-a concentration are identified as required ECV products. Time series of the products at the global scale and at high spatial resolution, derived from ocean-colour data, are key to studying the dynamics of phytoplankton at seasonal and inter-annual scales; their role in marine biogeochemistry; the global carbon cycle; the modulation of how phytoplankton distribute solar-induced heat in the upper layers of the ocean; and the response of the marine ecosystem to climate variability and change. However, generating a long time series of these products from ocean-colour data is not a trivial task: algorithms that are best suited for climate studies have to be selected from a number that are available for atmospheric correction of the satellite signal and for retrieval of chlorophyll-a concentration; since satellites have a finite life span, data from multiple sensors have to be merged to create a single time series, and any uncorrected inter-sensor biases could introduce artefacts in the series, e.g., different sensors monitor radiances at different wavebands such that producing a consistent time series of reflectances is not straightforward. Another requirement is that the products have to be validated against in situ observations. Furthermore, the uncertainties in the products have to be quantified, ideally on a pixel-by-pixel basis, to facilitate applications and interpretations that are consistent with the quality of the data. This paper outlines an approach that was adopted for generating an ocean-colour time series for climate studies, using data from the MERIS (MEdium spectral Resolution Imaging Spectrometer) sensor of the European Space Agency; the SeaWiFS (Sea-viewing Wide-Field-of-view Sensor) and MODIS-Aqua (Moderate-resolution Imaging Spectroradiometer-Aqua) sensors from the National Aeronautics and Space Administration (USA); and VIIRS (Visible and Infrared Imaging Radiometer Suite) from the National Oceanic and Atmospheric Administration (USA). The time series now covers the period from late 1997 to end of 2018. To ensure that the products meet, as well as possible, the requirements of the user community, marine-ecosystem modellers, and remote-sensing scientists were consulted at the outset on their immediate and longer-term requirements as well as on their expectations of ocean-colour data for use in climate research. Taking the user requirements into account, a series of objective criteria were established, against which available algorithms for processing ocean-colour data were evaluated and ranked. The algorithms that performed best with respect to the climate user requirements were selected to process data from the satellite sensors. Remote-sensing reflectance data from MODIS-Aqua, MERIS, and VIIRS were band-shifted to match the wavebands of SeaWiFS. Overlapping data were used to correct for mean biases between sensors at every pixel. The remote-sensing reflectance data derived from the sensors were merged, and the selected in-water algorithm was applied to the merged data to generate maps of chlorophyll concentration, inherent optical properties at SeaWiFS wavelengths, and the diffuse attenuation coefficient at 490 nm. The merged products were validated against in situ observations. The uncertainties established on the basis of comparisons with in situ data were combined with an optical classification of the remote-sensing reflectance data using a fuzzy-logic approach, and were used to generate uncertainties (root mean square difference and bias) for each product at each pixel.

## 1. Introduction

Visible spectral radiometry of the ocean (ocean-colour remote sensing) provides time series of digital chlorophyll fields (a measure of phytoplankton abundance) on synoptic scales. These data have stimulated a revolution in biological oceanography, as well as in Earth System science, and they have enabled many important advances [1].

Phytoplankton consume carbon dioxide through photosynthesis, being responsible for cycling some 50 GT of carbon globally on an annual scale [2]. They are also important players in the cycling of other elements in the ocean, including nitrogen, silica, and iron [3]. Phytoplankton are understood to impact atmospheric processes such as formation of aerosols through complex pathways [4,5,6,7]. Understanding phytoplankton is fundamental to anticipating how the marine ecosystem might respond to climate variability and climate change [8]. Phytoplankton absorb sunlight and are therefore implicated in modulating the distribution of heat in the surface layer of the ocean and, hence, in the air–sea exchanges of energy [9,10,11]. Because of these important processes, there is an imperative to monitor their dynamics at the global scale, with high resolution in time and space. Remote sensing of ocean colour provides our only window into phytoplankton dynamics and into the pelagic ecosystem on synoptic scales, hence its characterisation as revolutionary.

However, visible spectral radiometry has yet to realise its full potential for various reasons: high incidence of missing data; finite duration of individual missions; gaps in the data record; lack of continuity of missions; change of spectral characteristics of sensors used in successive missions with implications for consistency in data; and often, lack of error fields associated with the principal properties recovered by the standard methodologies.

Ocean colour has been recognised as an Essential Climate Variable (ECV) by the Global Climate Observing System [12,13]. In the context of climate research, where one searches for small climate-related signals embedded in data with a dynamic range of more than four orders of magnitude, sampled from a noisy environment, the data stream should have the requisite precision. The sampling should be sustained over several decades, given that the oceans are subject to variability at decadal and multi-decadal scales [14,15,16]. However, satellites and the sensors they carry have finite lifespans, typically about ten years. It is therefore required that data from different missions be joined in a seamless manner to produce a continuous time series that is free of artificial trends arising from blending disparate datasets. Any biases between sensor-specific products have to be removed. It is also required to maintain a minimum signal-to-noise ratio in the data and to associate each point in the record with an estimate of uncertainty.

Against this background, in 2010, the European Space Agency (ESA) launched its Climate Change Initiative (CCI, [17]), dedicated to producing climate-quality data for a suite of ECVs. The ocean-colour element (OC-CCI) was tasked to blend the existing major data streams for ocean colour into a coherent record meeting the requirements for climate-quality products. The resulting time series for the period 1997 to 2018 is the most internally consistent (all radiometric products band-shifted to a common set of bands corresponding to SeaWiFS (Sea-viewing Wide-Field-of-view Sensor)) and stable (corrected for inter-sensor bias) ocean-colour record available so far. It is complete with estimates of uncertainty and, thanks to a new procedure for atmospheric correction (POLYMER (POLYnomial based algorithm [18] applied to MERIS in all versions and to MODIS-Aqua in v3.1)), has fewer missing data than any other global time series for ocean colour. The focus of the work is on open-ocean (case-1) waters. Here, we report on the innovations used to achieve this goal, describing the resultant products and the implications for future work.

A key to notations and acronyms used in the paper is provided in Table 1.

## 2. User Consultation

The user community, including ecosystem modellers and remote-sensing scientists, was polled through an online questionnaire and phone calls as well as through targeted meetings at the beginning of the project [20], all of which together yielded responses from about one hundred scientists. A key point that emerged from the consultation was that the products identified by GCOS [12] (water-leaving radiances or remote-sensing reflectances at multiple wavelengths and chlorophyll concentration) might be considered only a minimal set: the user community was also interested in additional ocean-colour-derived products such as spectral inherent optical properties (IOPs, e.g., absorption and scattering coefficients of seawater and its particulate and dissolved constituents), primary production and phytoplankton community structure.

For the spatial and temporal resolutions of the chlorophyll product, there were various requirements depending on the user type (remote-sensing scientists or modellers) and on the geographic scope of research (global or regional). Accordingly, spatial resolution requirements ranged from 1 km or less for the remote-sensing scientists to 1${}^{o}$ for some modellers. Temporal requirements ranged from daily to monthly for both groups. In addition, users emphasised the importance of having uncertainty or error characteristics associated with each product. They also identified the need for sufficient stability in the products over the long term, necessary to distinguish between real trends in the data from artefacts introduced by lack of stability in data (e.g., 1–2% for chlorophyll-a concentration over 10 years).

Though the project focussed on open-ocean, case-1 waters, many users were interested in extending algorithms to allow retrieval from optically complex coastal waters (typically referred to as case-2 waters) or, failing that, at least to help identify the boundaries of case-2 waters.

Since users identified phytoplankton types as a desirable product and since many algorithms for discrimination of phytoplankton types require more than the blue and green wavebands that are typically used in empirical chlorophyll-retrieval algorithms, it followed that the remote-sensing reflectances provided should include longer wavebands in the visible than just blue and green wavebands. Many users also indicated that they would be interested in studying the phenology—the dynamics of phytoplankton at seasonal scales—and its inter-annual variability. This requirement implied that the incidence of data gaps should be minimised, since temporally complete data are required to extract accurately phenological indicators such as the time of initiation, the duration, and the time of peak of phytoplankton blooms.

Overall, the user community (biogeochemical modellers and remote-sensing scientists) requires long-term, stable, and gap-free products with well-characterised uncertainties and additional variables (inherent optical properties, phytoplankton functional types, phenology indicators, and particle size distributions). Some of these additional products would facilitate the extension of ocean-colour products from the open ocean to the coastal areas. The user requirements were taken into consideration when selecting the products to be included in the outputs generated, and they also influenced the criteria that were used to select processing algorithms [21].

The various steps involved in the product generation are described below and summarised in Table 2. The main protocols for product generation were established in the first version (v1). Subsequent versions brought various improvements to the product series, while retaining the conceptual framework introduced in v1. Therefore, v1 is described in more detail than the subsequent versions.

## 3. Data

All new satellite products presented in this study refer to those generated within the OC-CCI project until 2018 and published as OC-CCI dataset version 1 (OC-CCI-v1) and subsequent versions (now in OC-CCI-v4). Details regarding the processing chain of each version are given in Table 2. The table also provides links to the products and to processing tools.

### 3.1. Satellite Data

Four sets of satellite data were used: SeaWiFS (Sea-viewing Wide-Field-of-view Sensor; overpass at 12:00 local time; nadir resolution 4.5 km at Global Area Coverage or GAC mode and 1.1 km nadir resolution at Local Area Coverage or LAC mode); MODIS-Aqua (Moderate-resolution Imaging Spectroradiometer-Aqua, overpass at 13:00 local time; nadir resolution 1 km) from NASA (National Aeronautics and Space Administration) of USA; MERIS (MEdium spectral Resolution Imaging Spectrometer reduced resolution data with nadir resolution of 1.1 km; overpass at 10:00 local time) of ESA (European Space Agency) and VIIRS (Visible Infrared Imaging Radiometer Suite with a resolution of 750 m at nadir; equator crossing time at 1:30 p.m. local time), which is a joint NASA-NOAA (National Oceanic and Atmospheric Administration) mission. Of these, SeaWiFS operated from September 1997 until December 2010, and MERIS operated from March 2002 to May 2012. The MODIS-Aqua sensor was launched in May 2002 and is still operational at the time of writing the manuscript. The VIIRS sensor was launched in October 2011 and is currently operational. Only Global Area Coverage (GAC) data of SeaWiFS were used in v1 and v2. Local Area Coverage (LAC) data were added from v3.1 onwards. Level 1B data were used for all satellites.

Ancillary data on ozone and meteorological variables required for POLYMER processing came from the National Center for Environmental Prediction (NCEP) as implemented by NASA for ocean-colour processing. This ensured consistency in auxiliary data used in all sensors, since L2Gen uses the same set of auxiliary data in its processing chain.

### 3.2. In Situ Data

In situ data were used for three parts of the work: (i) for comparison of algorithms as part of algorithm selection process; (ii) for validation of satellite products; and (iii) for uncertainty characterisation of the products. The database was composed of in situ measurements of remote-sensing reflectances at multiple wavelengths, concentration of chlorophyll-a, inherent optical properties, and the diffuse attenuation coefficient. Data from the different sources were merged into a single unified database with the aim of increasing the number of match-ups available for validation and their distribution in time and space. To merge all data, methodologies for homogenisation, quality control, and removal of duplicate data were implemented. Homogenisation focused on the conversion of all data into a standard format with respect to units and nomenclatures. The in situ database associated with v2 of OC-CCI has been published [22] and that assembled for v4 has been submitted [23].

Radiometric observations were acquired originally from multiple sources after they had been quality-controlled, time-averaged, and extrapolated to the surface and, therefore, required only conversion to standard format. Remote-sensing reflectances, if not directly available, were computed from irradiance reflectance or water-leaving radiances. To ensure the highest consistency between in situ and satellite data, only exact or fully-normalized remote-sensing reflectances, which account for the bidirectional nature of the light field [28,29], were used. For inherent optical properties, absorption coefficients of phytoplankton and of coloured dissolved matter plus detritus, as well as those of backscattering by particles, were either assembled or computed from the available data. The diffuse attenuation coefficient for downwelling irradiance in water did not require any conversion and was compiled as originally acquired. For chlorophyll-a concentration, preference was given to measurements of total chlorophyll-a derived from HPLC (High Performance Liquid Chromatography) methods, which is calculated by summing all reported chlorophyll-a derivatives, including divinyl chlorophyll-a, epimers, allomers, and chlorophyllide-a. In the absence of HPLC measurements, chlorophyll-a concentration derived from fluorometric or spectrophotometric techniques, which was far more readily available, was used. Chlorophyll measured using in vitro techniques was given priority; therefore in vivo fluorometer measurements on profiling instruments or on continuous underway instruments were excluded whenever they were identified. All chlorophyll-a replicates (multiple measurements in a station) and multiple vertical observations found in the top 10 m were averaged to a single value if the coefficient of variation (CV) was less than 50%; otherwise, they were discarded.

The top 10 m of the water column was taken as representative of the surface layer, and best estimates of chlorophyll-a concentration for this layer were then compared with the satellite data. It is well known that, when the water column is vertically inhomogeneous, the chlorophyll concentration inferred from satellite radiometry would be close to a weighted average value of the first optical depth of the water column, with the weighting function depending on the optical properties of the water in that layer [30,31,32]. However, the optical properties of the water column and hence the geometric depth corresponding to the first optical depth, change with wavelength. In a multi-wavelength algorithm, it is not a trivial problem to determine the relevant optical depths and the implications for satellite algorithms (see, for example, Sathyendranath and Platt [31]). When multiple algorithms are used in the generation of products, as is the case with the recent OC-CCI products, the problem of using appropriately weighted in situ chlorophyll concentrations in validation exercises quickly becomes intractable. Further, the more esoteric the validation procedure, the smaller the community that could potentially appreciate what exactly the product represents in relation to in situ observations. These considerations led to our choice of a simple average chlorophyll concentration for the top 10 m of the water column for validation of the OC-CCI chlorophyll product. We recognise, however, that this choice invites the risk of increasing the apparent differences between satellite observations and in situ observations. This being the case, the validation exercises undertaken in this study are best interpreted as testing how well the satellite data represent the surface chlorophyll values.

**Figure 1:**
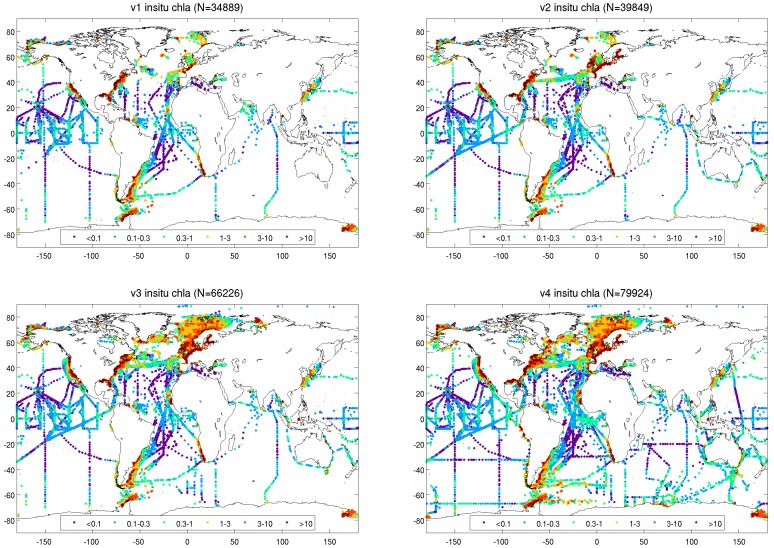
Global distribution of in situ chlorophyll data assembled for the four versions of OC-CCI: The colours are indicative of the values of the variables (see the colour bar); units of chlorophyll: mg m${}^{-3}$.

Quality checks on the radiometric and pigment data included visualisation of data through time series and maps and the removal of observations with unrealistic values, missing time or geographic coordinates and fields, poor quality (as indicated by flags in original databases), inappropriate method of observation, and suspicious observations (e.g., spuriously high or low). Duplicate data present in multiple databases were identified and removed on the basis of the metadata (e.g., experiment or cruise name) and temporal-spatial thresholds. The metadata of each in situ measurement (source, cruise or experiment, and principal investigator) were propagated throughout the processing into the merged database. The final merged database represents the output of an attempt to gather, homogenise, and merge several data sources that have emerged from the long-term efforts of the ocean-colour and biological oceanographic communities to provide scientists with high-quality in situ observations. As an example, geographic distributions of chlorophyll concentrations in the in situ database associated with the four versions of OC-CCI products are shown in Figure 1.

For further details regarding the sources of the original data and the process of quality checking and elimination of duplicates, please refer to Valente et al. [22] and Valente et al. [23].

### 3.3. Match-Up Database

The in situ data were matched with corresponding satellite data for use in algorithm selection, for validation of the products, and for generation of uncertainties. The match-ups were identified by the nearest latitude and longitude, the central pixel being co-located with each of the in situ data. The surrounding pixels (3×3 box, with the in situ datum in the centre) were selected for further analysis. Only those pixels that had a valid central pixel and which satisfied various product-specific checks (e.g., chlorophyll within the 0.01 to 100 mg m${}^{-3}$ range and water depth greater than 50 m according to bathymetry) were selected for further analysis. As the OC-CCI product is a daily merged product of MODIS-Aqua, MERIS, SeaWiFS, and VIIRS, with overpass between ∼10:00 and ∼13:30 local solar time, the acceptable time window for match-ups was initially set to 09:00–15:00, but subsequent tests showed minimal significant effect from accepting in situ samples at any time of the same day; the time window was therefore set to same day. Homogeneity criteria (e.g., CV < 0.15; at least five valid pixels in the 3×3 box) were used to test the impact of non-homogeneity in the vicinity of the match-up data on the uncertainty estimates. However, finally, all data with valid central pixels were used for uncertainty characterisation in the interest of maximising the number of observations and geographical and seasonal coverage of the match-up database. Statistical information on the 3×3 box, including mean, median, and coefficient of variation, was retained in the match-up database for further downstream analysis.

## 4. Algorithm Selection Criteria

A comprehensive set of criteria was established for selection of atmospheric-correction processors and of in-water bio-optical algorithms, as described in Brewin et al. [33], Müller et al. [34], and Müller et al. [35]. The criteria included a quantitative assessment and a qualitative assessment of the suitability of algorithms for climate studies [21]. The quantitative assessment consisted of a comparison of algorithm outputs with corresponding in situ data for both the in-water algorithms and the atmospheric correction processors. Furthermore, the homogeneity of angular dependencies (i.e., dependency on viewing geometry) of the various atmospheric correction processors was also studied.

### 4.1. Objective Scoring System Based on Quantitative and Qualitative Criteria

The goal was to generate products for use in climate studies; therefore, suitable processors and algorithms that best met the requirements of climate studies, as defined by the user community, had to be selected.

Whereas the round-robin comparison treated the atmospheric correction processors and in-water algorithms separately, both approaches were based on similar quantitative and qualitative assessments of algorithm performance. The quantitative criteria for the assessment of atmospheric correction processors and in-water algorithms are described in detail in Müller et al. [34] and Brewin et al. [33], respectively. The semi-quantitative assessment conducted to test the homogeneity of products over the full swath of a satellite image and the dependencies of the atmospheric correction processors on the satellite viewing geometry (angular dependencies) are described in Müller et al. [35]. These are summarised below.

#### 4.1.1. Quantitative Criteria

For the quantitative comparison, match-up datasets were constructed first. To test the atmospheric correction processors, a match-up database of remote-sensing reflectances ($R_{rs}$) derived from satellite and in situ observations was created. To test the in-water algorithms, an in situ database with inherent optical properties, diffuse attenuation coefficient, and chlorophyll concentration was matched with corresponding $R_{rs}$ from satellite data.

The geographic distribution and trophic conditions corresponding to observations in any match-up database are unlikely to be representative of the entire global ocean over a full seasonal cycle. Therefore, in both the atmospheric-correction and in-water inter-comparisons, the method of bootstrapping [36,37] was used to explore the effect of the in situ datasets on the results. This involved randomly resampling, with replacement, the in situ data to create several thousand of new datasets, each of the same size as the original dataset. The quantitative statistical tests and the scoring method, developed for atmospheric-correction and in-water comparisons, were run for each new dataset to explore the impact of choice of dataset and to account for uncertainties in the methodology adopted for scoring.

For both the atmospheric-correction and in-water inter-comparisons, the performance of the candidate processors and algorithms was evaluated using a suite of statistical tests commonly used in comparisons between modelled and in situ data [38,39]. For comparisons of algorithms, these statistical tests were employed to develop an objective methodology designed to rank the quantitative performance of the candidate algorithms and processors. The objective methodology differed slightly with respect to the in-water and atmospheric-correction comparisons due to differences in the number of algorithms tested, differences in the number and types of variables, and inherent differences in the implementation of atmospheric-correction and in-water algorithms, details of which can be found in Müller et al. [34] and Brewin et al. [33]. The comparisons were designed to rank the performance of the algorithms objectively and have the potential to be implemented routinely, such that the performance of emerging algorithms could be compared with that of earlier algorithms. Such systematic comparisons could be helpful to identify more suitable algorithms as they emerged.

#### 4.1.2. Qualitative Criteria

Algorithm selection depends not only on the quantitative performance but also on the suitability of the algorithm for the applications envisaged and on the user requirements. For climate-change studies, these include the ability of the algorithm or processor to create a long-term, consistent, uncertainty-characterised time series of ocean-colour products; to create products that best suit the requirements of the user community; to quantify a variety of properties of the marine ecosystem that are relevant to climate studies and accessible from satellite ocean-colour data; and to be robust against potential modifications in the marine ecosystem in a changing climate. Ideally, the most appropriate algorithm would meet all these requirements and compare well in statistical tests of performance. Sathyendranath et al. [21] have discussed the ideal characteristics of ocean-colour algorithms selected for climate-change studies and examined the extent to which OC-CCI products meet them. In the work presented here, algorithms (both atmospheric-correction and in-water) were evaluated using a set of questions constructed to ascertain the extent to which they were suited to climate-change studies. Several selection criteria were considered including, for the atmospheric-correction processors, the applicability of the processor to all sensors, open access to an algorithm theoretical baseline document (in the interest of transparency and traceability), and coverage obtained (a highly-performing algorithm that yielded limited geographical coverage was not deemed suitable). Although consistency in atmospheric correction across all sensors was recognised as being important, quantitative performance of the algorithm for each sensor was ranked higher in the decision hierarchy than inter-sensor consistency between processors: the reasoning was that consistency in method need not yield consistent quality in product when the sensors differ from each other, as is the case here. For in-water algorithms, the robustness of the algorithm in the event of potential modifications to the marine ecosystem in a changing climate was considered important. For both atmospheric-correction and in-water algorithms, it is also important to be able to apply the algorithm to the next generation of ocean-colour sensors. A subjective scoring system was applied to the qualitative criteria for ranking the algorithms.

## 5. Atmospheric Correction

All the satellite data were processed using all the processors available and compared with the corresponding in situ data. A set of statistical tests was used to assess the performance of the atmospheric correction at each wavelength, and a $\chi^{2}$ test was also used to evaluate the spectral quality of the retrieved $R_{rs}$ signal. The results were translated into objective scores, and the analysis was repeated using bootstrapping methods, as described above [34].

As a complement to the comparison of satellite data with in situ measurements, systematic sensor-dependent behaviour of satellite products across the satellite-sensor track (i.e., along scan) was studied [35]. The particular focus of the investigation was dependence of products on the position of pixels across track. For example, the sun-zenith angle and the sensor viewing angle are related to the along-scan position of a pixel. Systematic effects across track could correspond to incomplete or improper accounting for geometric effects (in particular, during normalisation or smile correction applied to account for small shifts in the central wavelength of bands across a scan line) in the atmospheric correction processor. These residual effects can distort time-series results at fixed locations. Angular dependencies were tested on MERIS data from the South Pacific Gyre and the North Atlantic regions for four atmospheric-correction processors.

On the basis of the round-robin analyses, the POLYMER atmospheric-correction processor [18] was selected for processing MERIS data and the NASA L2Gen processor was selected for SeaWiFS, MODIS-Aqua, and VIIRS data (see Table 2). An exception is v3.1, where POLYMER was selected for MODIS-Aqua in addition to MERIS. Similar round-robin match-up analyses were carried out prior to generation of each of the subsequent versions of OC-CCI products. An exception is v4: it is an update to v3.1, produced with the latest reprocessing of MODIS-A, SeaWiFS, and VIIRS by NASA in 2018, which accounts for some sensor drift in recent years. Since the full processing chain for atmospheric correction, in particular for the part dealing with system vicarious calibration, was available only for the L2Gen processor of NASA at the time of product generation, it became the default processor for these three sensors in this instance, while POLYMER was retained for MERIS processing.

System Vicarious Calibration (SVC) is recognised as a key element of the ocean-colour processing to reach the requirements of Climate Data Record: radiometric uncertainty less than 5% in the blue and green spectral regions [40]. This consists in the calibration of the system comprising both the sensor (Level 1B data) and the Level 2 processing chain through the combination of i) high-quality sea-truth water-leaving radiance and ii) atmospheric models used in the atmospheric correction process [41]. For L2Gen processing, the SVC gains were the ones provided by NASA following the Franz et al. [42] approach, based on measurements of the Marine Optical BuoY (MOBY, Clark et al. [43]) at each of the sensor bands. For POLYMER processing, this standard approach could not be applied because of the spectrally coupled inversion of the whole spectrum. The SVC gains for POLYMER were set to unity for all spectral bands in the initial v1 processing. The POLYMER gains in the subsequent versions were computed by a dedicated method developed in the OC-CCI project [44]. This method computes the SVC gains by minimizing the discrepancy between the MOBY data and the POLYMER retrieval at sea level, implicitly taking into account the spectral constraints of the marine and atmospheric models embedded in the atmospheric correction procedure.

## 6. Pixel Identification

The objective of the pixel classification component of the processing chain is to determine whether a satellite measurement is suitable for further processing and, thus, to be included in the time series for climate studies. Measurements over land, sea ice, or thick clouds are obviously unsuitable cases. Less obvious are cases with a semi-transparent atmosphere, e.g., due to a thin cirrus or high aerosol optical thickness and pixels which have sub-pixel variability due to partial land or cloud cover, cloud shadow, high glint reflectance, or white caps. Operationally, the adjectives “semi-transparent”, “thin”, or “high” are imprecise; in practice, the pixel identification is tied to the capability of the atmospheric-correction algorithms to treat individual non-clear-sky water pixels. Hence, for MERIS data, it was important to identify pixels that were treatable using the POLYMER processor. Another possibility is that the surface of the ocean or the atmosphere or the viewing geometry might be outside the scope of the processing algorithm. Examples are aerosols which are not included in the aerosol model suite used for atmospheric correction or exceptional plankton blooms which are not included in the bio-optical model for water. Such observations should not be processed further and should be identified by the pixel classification.

The requirement for Level 3 products in climate studies introduces an important constraint for pixel classification: the classification has to be clear-sky conservative, i.e., every pixel classified as being good for further processing must have a high probability of having been correctly classified. At the same time, in the interest of minimising gaps in data, too many pixels should not be misclassified as being unusable. The cloud screening for MERIS provides three levels of clouds: opaque, semi-transparent, and clear sky. We optimised the distinction between the three classes by analysing the increase in the error on match-ups. All data generated at Level 2 are processed automatically to Level 3, and only good pixels can be taken forward at this stage to meet the accuracy requirements for climate-change studies. Pixels considered good are those not flagged as clouds (including semi-transparent clouds for MERIS) or as sea ice or floating vegetation and where the quality flags provided by the respective atmospheric correction algorithms permitted usage of the retrieved marine reflectance.

In this work, a new pixel-classification algorithm (Idepix) was implemented for MERIS data processing in v1 and for MERIS and SeaWiFS in v2, with L2Gen being used for the other sensors. The unusable pixels detected included three types of pixel classes: cloudy pixels, sea ice pixels, and pixels of mixed surface types, that is, those which include portions of land in their footprints. In subsequent versions, a combination of NASA pixel classification algorithm in L2Gen and Idepix was retained.

### 6.1. Cloud Screening

The cloud screening implemented here is a combination of the spectral threshold method and classification methods based on feature extraction, including spatial structure [45,46]). It uses properties, such as brightness or whiteness, which are derived from spectral tests, and also abstract properties generated from statistical analysis of the data, for example, by training a neural network. For each property used, an expression is defined to calculate a normalised value within the range 1 (no cloud) to 2 (totally cloudy). The link between the normalised values and the outputs from POLYMER was studied using match-ups in the OC CCI database to identify the range of values for which the error in atmospheric correction was too high. The final classification of a given pixel is an arithmetic combination of the normalised values derived for each property used.

### 6.2. Sea-Ice Detection

Sea ice can have many colours from the bright-white of snow-covered ice to the brown-green of old ice and different shades of blue. In most cases, it is snow-covered and looks similar to clouds and can be differentiated easily with a spectral band at 1.6μm because of the stronger absorption of snow compared with clouds in the infrared region. Unfortunately, MERIS does not have such a SWIR (short-wave infrared) band. In its absence, the two nearest bands outside the water–vapour absorption bands, namely band 13 (865 nm) and 14 (885 nm), are used here. The difference in $R_{rs}$ at these bands after Rayleigh correction, normalised to the sum of the two values, $\left(R_{rs}\left(885\right)-R_{rs}\left(865\right)\right)/\left(R_{rs}\left(885\right)+R_{rs}\left(865\right)\right)$, with a threshold value of 0.014, is used as a snow index to discriminate clouds from snow and ice. The snow index applies only if a pixel is bright, and the brightness feature of the cloud screening is used for this purpose.

### 6.3. Mixed-Pixel Identification

A mixed pixel refers to a mixed spectrum that is the combination of the individual spectra of each component within the pixel, weighted by the areal proportion applicable to that component. Near the coast, pixels can be affected by several kinds of surfaces (land, water, bottom reflectance, floating vegetation, and intertidal areas), and surface plankton blooms (scum) can occur offshore as well. All these are considered as mixed pixels. There is a need for screening pixels in the sub-pixel level and for identification and flagging of mixed pixels because they can disturb the spectral signal significantly. Scum identification has not been attempted here because of difficulties with generic implementation (see example of a specific implementation in Kahru and Elmgren [47]).

For the mixed pixel analysis, end member spectra (spectra corrected for gaseous transmission and Rayleigh scattering) have been defined for typical water, land, and cloud spectra for the bands at 560, 665, 709, 753, 778, and 865 nm, normalised by the sun-zenith angles to account for variations with the viewing and illumination angles, and the observed spectrum at each pixel is assumed to be a linear combination of the different end members using spectral mixture analysis [48]. The contribution from each end member to the pixel spectrum is computed using a fully constrained linear spectral un-mixing algorithm. Several threshold tests on the contributions, in conjunction with spectral ratio threshold tests and other pixel masks (coming from the cloud screening), are used to generate the mixed-pixel flag.

### 6.4. Validation of Pixel-Identification Algorithm

A large dataset of manually classified MERIS pixels was collected (PixBox Dataset). A set of attributes was defined, allowing each of the manually selected pixels to be classified by visual inspection. These attributes included totally cloudy; clear sky over water; snow or ice; land; non-clear sky over water; snow or ice; and land. Spatially mixed pixels included cloud over land; partial snow or ice over water; snow or ice over land; and snow or ice over water. Several additional attributes were assigned as appropriate, such as very turbid or floating vegetation. The manual selection was performed by an experienced scientist. The attributes were assigned partly from static background maps or for particular dates, times, and locations. Most attributes, however, were manually assigned by the sampling expert. The PixBox dataset was taken as the reference against which the automated classification was validated.

The unambiguous pixel types are ocean pixels under clear skies, as well as totally cloudy, snow-covered, or ice-covered pixels. The PixBox dataset was constrained to oceanic and coastal waters, i.e., rivers and inland waters were excluded. The confusion matrix (Table 3) shows that the cloud cases were identified correctly by the classification in nearly all cases (15,068 from 15,157 = 99.4%). However, a significant portion of the clear sky water pixels were misclassified as clouds (1033 from 6468 = 15.9%), for example, in the coastal zone where bright water can be found. This behaviour is a result of the cloud-screening strategy, which is clear-sky conservative: in doubtful cases, a pixel was removed from further processing to reduce the risk of bad pixels affecting the final products. In further analyses, many test scenes were processed and the performances of Idepix and L2Gen pixel identifications were compared. If appropriate, a combination of L2Gen and Idepix was used for pixel masking.

This procedure, established in the course of developing v1, has been progressively refined in subsequent versions. When implementing pixel masks and flags, one has to weigh the need for high-quality data against the need for high coverage. When creating a climate-quality dataset, the temptation to let suspect data through in the interest of improved coverage has to be resisted. Therefore, in each subsequent version of OC-CCI, we have continued to improve Idepix methodology, to test the outputs, and to compare the results with the L2Gen flags and masks. When in doubt, we have chosen to be conservative and to apply all standard NASA flags to filter L2Gen-processed images rather than to risk contamination of products by poor-quality data. A case in point is the NASA stray-light flag, which impacts coverage significantly, but careful examination of many test scenes showed that ignoring this flag allowed dubious $R_{rs}$ values to pass through. Therefore, stray-light flag has been applied to OC-CCI products for all sensors processed using L2Gen and in v3.1; these flags were also implemented on POLYMER-processed MODIS-Aqua data. Note that all in-water products derived from $R_{rs}$ spectral values are restricted to those pixels where the quality of $R_{rs}$ passed all the appropriate tests for quality, ensuring a set of products of consistent quality that also meet the requirements of transparency and traceability.

### 6.5. Additional Filters and Post-Filters

In some or all the versions, the well-verified data processor L2Gen packaged as part of SeaDAS was used for processing SeaWiFS, MODIS-Aqua, and VIIRS. Some additional post-filtering was done. Primarily, any pixels with negative $R_{rs}$ values in the 412–560 nm range were discarded, as they would probably have resulted from a flawed atmospheric correction. The 670 nm band was exempted because negatives close to zero are common at this wavelength due to low signal levels; instead, these values were trimmed to zero and the pixels were retained if all other bands were positive. Some other range filters were applied, removing impossibly high reflectances (e.g., due to unfiltered glint) or chlorophyll well outside the range of case-1 waters that OC-CCI targets. Other than this, there were no filters applied to mask potential case-2 (optically-complex) waters.

## 7. Band Shifting

OC-CCI produces a consistent multi-mission data record of normalised remote-sensing reflectance $R_{rs}$, initially using data from the MODIS-Aqua, SeaWiFS, and MERIS missions, to which VIIRS data were added subsequently (see Table 2). These four missions have all produced multi-spectral $R_{rs}$ data but with different sets of bands, which is an obstacle for inter-comparison and merging. Whereas some central wavelengths coincide approximately, others differ by more than 5 nm. The issue of different bands is also relevant in validation activities, since multi-spectral field observations do not necessarily coincide with the satellite values in their spectral character.

An approach was developed here to correct for band differences, a process referred to as band shifting. Band-shifting schemes have been developed for validation analyses, with some of these approaches relying on relationships proposed for clear open-ocean waters or for specific regions (e.g., [49,50,51,52]). The approach developed here is a more general one, based on the Quasi-Analytical Algorithm (QAA) [19], and was devised to map all the normalised $R_{rs}$ values on to the SeaWiFS wavelengths (412, 443, 490, 510, 555, and 670 nm) for the global application required in this work. Its main characteristics are summarised here: more details are provided by Mélin and Sclep [53]. Note that band-shifting and bias correction (described in the next section) are applied to normalised remote-sensing reflectance, representative of nadir-viewing conditions.

The bio-optical algorithm QAA performs the inversion of the $R_{rs}$ spectrum to derive inherent optical properties (IOPs) at 443 nm, yielding the coefficients of backscattering by particles $b_{bp}$ and absorption by phytoplankton $a_{ph}$ and by coloured detrital matter (including coloured dissolved organic matter or gelbstoff and particulate detrital matter) $a_{dg}$. To perform the band shifting from an input wavelength $\lambda_{i}$ to a target wavelength $\lambda_{t}$, the IOPs are calculated at these two wavelengths using the spectral shape assumed in the QAA for $b_{bp}$ and $a_{dg}$, and that given by Bricaud et al. [54] for $a_{ph}$ (since the QAA does not make assumptions on the spectral shape of $a_{ph}$, except for the ratio of $a_{ph}$ at two wavelengths in the blue). Then, $R_{rs}$ values are estimated with the QAA run in forward mode, producing $R_{rs}^{f}\left(\lambda_{i}\right)$ and $R_{rs}^{f}\left(\lambda_{t}\right)$ (with the superscript *f* standing for forward and *t* standing for target). Finally, the estimated $R_{rs}$ at the target wavelength $\lambda_{t}$ is equal to $R_{rs}^{f}\left(\lambda_{t}\right)\cdot R_{rs}\left(\lambda_{i}\right)/R_{rs}^{f}\left(\lambda_{i}\right)$. This normalisation is necessary to ensure that the estimated $R_{rs}$ is equal to the input $R_{rs}$ if $\lambda_{t}=\lambda_{i}$ (which is not guaranteed, since $a_{ph}$ is not a QAA output at that wavelength).

To express $R_{rs}$ at 510 nm for MODIS-Aqua, band shifting is operated twice from 488 to 510 nm, and from 531 to 510 nm. Then, the final estimated value at 510 nm is the weighted average of the two resultant values. The performance of the QAA appears degraded for long wavelengths, particularly for $a_{ph}$ in the red, whereas it appears reliable at 443 nm [33,55], supporting the choice of that wavelength as a reference. In general, the selection of the QAA for operating the band shifting has the merit of being consistent with the bio-optical algorithm selected here for generating IOPs from satellite data ([33] see Section 4.3). This band-shifting scheme is also used with specific adaptations in the validation activities.

The performance of the band-shifting scheme has been assessed using hyper-spectral data from radiative transfer simulations and field observations [53]. The errors in $R_{rs}$ at the target wavelength are within a few percent, usually well below 5%. Another assessment has been performed with satellite data [53,56]. From global products on a one-twelfth-degree resolution grid, all coincident SeaWiFS and MODIS-Aqua daily $R_{rs}$ values were accumulated for the year 2003 and compared (approximately 50 million spectra). After band shifting, the median ratio μ between MODIS-Aqua and SeaWiFS data is closer to 1 in all cases. Thus, μ between MODIS-Aqua $R_{rs}$ at 488 nm and SeaWiFS $R_{rs}$ at 490 nm is 1.030 versus 1.007 after band shifting, i.e., when MODIS-Aqua $R_{rs}$ is expressed at 490 nm. Similarly, μ is 1.108 for comparisons between MODIS-Aqua data at 547 nm and SeaWiFS $R_{rs}$ at 555 nm, whereas it is 0.978 after band shifting. At 510 nm, the median ratio is also close to 1 (μ=0.979) despite the spectral distance between 510 nm and the original MODIS-Aqua bands (approximately 20 nm). On the other hand, improvements are fairly limited in the red domain (μ=0.92). The values of $R_{rs}$ in this spectral interval are often very low, and relative uncertainties are likely to be high. The results obtained with satellite data appear satisfactory, taking into account that the errors associated with the band-shifting scheme cannot be separated from actual differences between sensor-specific products.

## 8. Bias Correction and Merging

The strategy adopted in OC-CCI involves the production of a merged data set of $R_{rs}$ from the main ocean-colour data streams, currently SeaWiFS, MERIS MODIS-Aqua, and VIIRS, though initial versions did not include VIIRS (see Table 2). Besides the creation of a unified series of this ECV, it potentially removes the need for product-specific merging procedures applied to each derived product such as chlorophyll-a. Multi-mission $R_{rs}$ data records have already been used as inputs to merging schemes [57] or even produced as merged products [58,59,60]. The project specifically addresses climate research. Therefore, with respect to other activities dedicated to producing merged ocean-colour data, it should fulfil the additional requirement that the merging process should not introduce or should at least minimise spurious temporal artefacts resulting from inter-mission differences. These have been shown to vary significantly with time (mainly seasonally), space, and wavelength [50,56,61,62,63]. In the operational context discussed here, a simple and robust strategy has been adopted to address the latter two points, i.e., correcting for multi-year averaged biases computed for each grid point and each $R_{rs}$ band.

Specifically, multi-annual averages of $R_{rs}$ expressed at the SeaWiFS wavelengths (i.e., band-shifted $R_{rs}$ in the case of MODIS-Aqua, MERIS, and VIIRS) are computed over the period of 2003–2007, serving as the reference period when the three missions all functioned in an optimal way without any data gaps. In practice, 4-km daily data are first averaged as monthly composites, which are then combined into a monthly climatology for the period (i.e., all five January composites combined and so forth). Finally, these twelve monthly maps are averaged as a multi-annual (five-year) composite. This approach was chosen to reduce the impact of unusual or annual variations on the average and to minimise data gaps. The multi-annual bias of the average $R_{rs}$ values ($<R_{rs}>$) from mission *i* with respect to a reference mission (denoted as ref) is thus expressed as follows:

(1) $$\delta_{i}^{ref}\left(\lambda ,b\right)=\frac{<R_{rs}^{i}\left(\lambda ,b\right)>}{<R_{rs}^{ref}\left(\lambda ,b\right)>},$$

for wavelength λ and bin location *b*. The bias correction is the reverse operation applied on any daily $R_{rs}$ record at bin location *b* to produce a bias-corrected $R_{rs}$:

(2) $$R_{rs}^{i,corr}\left(\lambda ,b\right)=\frac{R_{rs}^{i}\left(\lambda ,b\right)}{\delta_{i}^{ref}\left(\lambda ,b\right)}.$$

The bias correction is treated as a ratio, which has the advantage of avoiding negative values that could result from considering the bias as an arithmetic difference. The mission selected as reference in the context of bias correction is SeaWiFS.

The MERIS, MODIS-Aqua, and VIIRS $R_{rs}$ series are thus brought into line with the SeaWiFS record. In the case of VIIRS, since there was no overlap with SeaWIFS, the band shifting was implemented by shifting first to MODIS-Aqua bands as an intermediary step. Therefore, the OC-CCI record for the first five years (1997–2002), the period for which SeaWiFS is the sole data source, is the SeaWiFS record unaffected by bias correction.

Following bias correction, merging is simply averaging the available data for a given pixel (SeaWiFS data and bias-corrected MERIS, MODIS-Aqua, and VIIRS data). Although the number of actual observations within a pixel for each sensor was propagated through the processing chain, it was not used as a weighting factor in merging, as it would have made SeaWiFS contribution small, if not negligible, compared with those of the three other sensors (4-km SeaWiFS GAC would provide one or two observations per 4 km pixel, whereas 1 km MODIS-Aqua or MERIS would provide up to 16). Instead, the merging used average data for each bin for each sensor, such that each sensor available was given equal weight.

In the initial version of OC-CCI, bias correction was applied as a single annual average value. In subsequent versions, the correction was applied using daily, multi-year running averages to eliminate any seasonal components in the bias correction.

**Figure 2:**
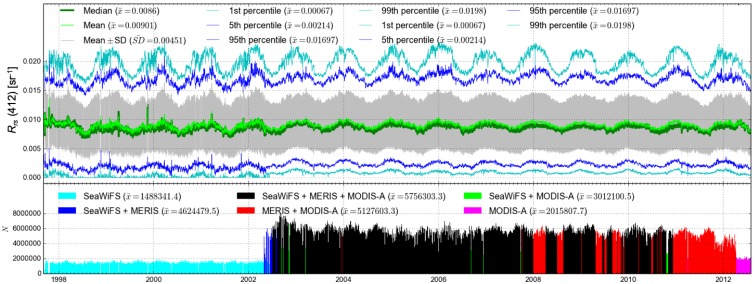
Top panel: Time-series values of global average $R_{rs}$ (412) [sr${}^{-1}$] and associated statistics (± one standard deviation; 1, 5, 95, and 99 percentiles for v1 dataset). Bottom panel: Number or pixels with valid data on a daily basis from 1997 third quarter to end of 2012. The colour code indicates the combination of sensors (SeaWiFS, MERIS, and MODIS-Aqua) contributing to the data stream. Note that the number of observations available per pixel more than triples on average, when MODIS-Aqua and MERIS data are added to SeaWiFS data.

Albeit simple, bias correction allows the effective removal of the average bias for each grid point and wavelength and guarantees a certain level of consistency for the merged $R_{rs}$ product. As an illustration, Figure 2 shows the average series of $R_{rs}$ at 412 nm accompanied by the number of samples included in the data stream. The first part of the record, when SeaWiFS was the sole data source (up to April 2002), is somewhat noisier with some isolated spikes, whereas the subsequent series is smoother. This is associated with a manyfold increase in the number of samples per day, as MERIS and MODIS-Aqua start contributing to the merged data set. However, the merged time series appears remarkably stable in time with no artefact associated with the varying availability of each data source, as is required in the context of climate research. Note that we refer here to stability in the data stream when new sensors come online and when old sensors cease to contribute without prejudice regarding any real temporal trends in the data. Since the bias correction is a single inter-sensor correction per location based on a comparison of the 2003–2007 period, the stability of the time series also provides some confidence, indirectly, in the quality of the temporal calibrations applied to each of the missions.

## 9. Generation of Optical Classes

Once the band-shifted, bias-corrected, merged, multi-sensor $R_{rs}$ values were generated at the SeaWiFS wavelengths, each pixel on each day was classified optically, using a fuzzy-logic approach [64,65,66]. In v1, the optical classes used were those provided by Moore et al. [64] (2009), with supplementary classes for coccolithophores provided by Moore et al. [65] (2012). In all subsequent versions, optical classes were custom generated using representative $R_{rs}$ spectra sampled from the corresponding $R_{rs}$ product for that version, as explained in Jackson et al. [66], with the coccolithophore classes from Moore et al. [65] retained in all versions.

## 10. In-Water Algorithms

Among the algorithms for chlorophyll retrieval that were tested (mostly designed for case-1 waters), empirical algorithms performed better than the semi-analytical ones. Because of the historical and heritage values of the NASA algorithm referred to as OC4v6 (a polynomial fit to maximum of three band ratios of remote-sensing reflectance), coupled with its strong performance in quantitative tests, it was selected as the algorithm of choice for chlorophyll retrieval in v1 and v2. In v3.1, algorithm performance was tested and the best algorithms were selected for each optical class [66].

Although they have not been identified as required products associated with the ocean colour ECV, it was decided to include inherent optical properties (IOPs) and apparent optical properties (AOPs) in the product suite, based on the feedback from the users. They are essential, for example, in eventual implementation of some algorithms for identification of phytoplankton functional types. The IOPs included are the total absorption coefficient and its components, which are attributed to: phytoplankton; detritus and yellow substances (combined); and total backscattering including contributions from pure seawater and from particles in suspension. The Quasi-Analytical algorithm (QAA) of [19,67] was selected for producing the IOPs. The AOP included in the time series is the diffuse attenuation coefficient at 490 nm, for which the model of Lee et al. [68] was selected. It uses the total absorption coefficient and the total backscattering coefficient derived from QAA and the solar-zenith angle as inputs. The algorithm is based on theoretical simulations and validations against observations and performed best in the quantitative comparison of algorithms [33].

The algorithm comparison demonstrated that all algorithms tested had some desirable features and also that other algorithms sometimes outperformed the selected algorithms in particular tests. Therefore, there is scope for further improvements to the algorithms, possibly by exploiting systems such as the Generalised Inherent Optical Property framework [69]. When the same data used for development of an algorithm are used also for validation of that algorithm, its performance may appear better relative to another algorithm for which independent data are used for algorithm development and validation. In the comparison carried out, it was difficult to evaluate the impact of independence or non-independence of the in situ dataset used for testing algorithm performance because the development of all algorithms was influenced to a degree by the in situ dataset used here. We tried to address this problem to some extent by using bootstrapping.

The strategy of generating time series for climate studies has to be open to the possibility that better algorithms may emerge in the future and should include periodic re-evaluations of algorithms, adoptions of new algorithms, and reprocessing of the data archive, when necessary.

## 11. Uncertainties: Root Mean Square Differences, Bias, and Relative Error

**Figure 3:**
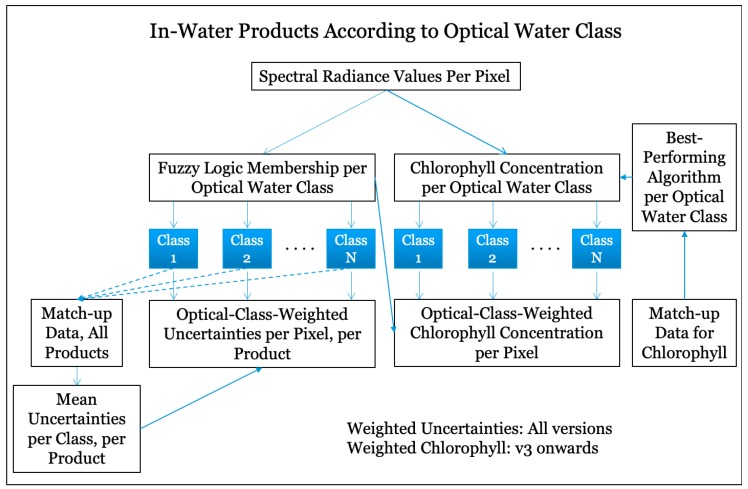
Schematic diagram showing how fuzzy-logic-based optical classification is used to propagate uncertainties in OC-CCI products on a per-pixel basis: From v3 onwards, optical classification has also been used to estimate chlorophyll concentration according to optical classes [66]. Note that this method could be extended to all products, provided sufficient data were available in each optical class for adequate uncertainty characterisation.

Uncertainty characterisation is a very useful and distinctive feature of all CCI products [70], though the specific approaches to uncertainty characterisation vary from one ECV to another. The user consultation carried out at the beginning of the OC-CCI project indicated an overwhelming preference for uncertainty characterisation based on comparison with in situ data. At the same time, CCI required that product uncertainties be specified on a per-pixel basis. Given the paucity of match-up data, uncertainty characterisation on a pixel-by-pixel basis was a challenge, which was addressed as follows: First, the OC-CCI in situ data described in Section 3.2 were matched with concurrent satellite observations for all OC-CCI products. For each pixel with match-up data point, the satellite-derived $R_{rs}$ spectrum was analysed to determine the memberships of the different optical classes in that pixel. This allowed us to calculate uncertainties (bias and RMSD) for each optical class, with the fuzzy-logic membership of each class for each match-up observation providing a weighting factor for calculating the mean uncertainty per optical class and for each product. Finally, uncertainties were assigned to each pixel in each image, according to per-class mean uncertainties, with the membership of the classes at the pixel providing a weighting function for the calculation of uncertainties. The process is shown schematically in Figure 3. Further details are provided in Jackson et al. [66]. Each of the products in the ocean-colour ECV product suite (except the backscattering coefficient) has uncertainties (bias and RMSD) assigned to every pixel. Uncertainties could not be calculated for the backscattering coefficient because of insufficient match-up data, but this lacuna will be addressed in the future as more data become available.

**Figure 4:**
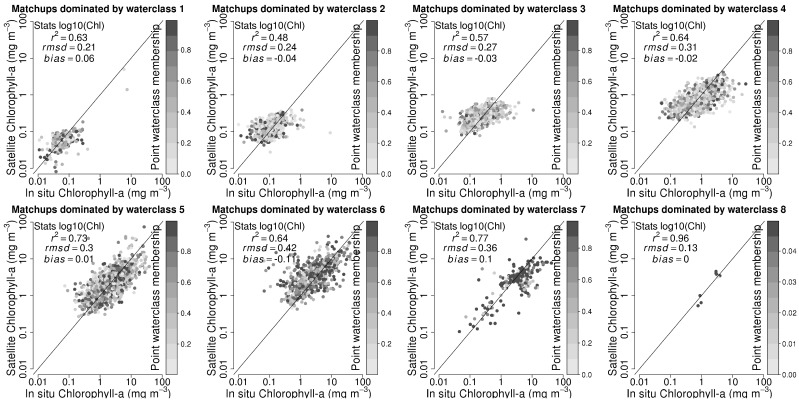
Comparison of in situ chlorophyll data with corresponding daily, merged satellite data for the eight water classes used in the study, for v1: The grey scale indicates the membership of water class in pixel. Note the change in grey scale for optical class 8, which has low membership values for the match-up observations. In the computation of RMSD and bias for each class, $\log_{10}$ chlorophyll was used and the observations were weighted by the membership. The overall statistics for chlorophyll and $R_{rs}$ values are provided in Table 4 for all versions.

As an example, Figure 4 shows how satellite-derived chlorophyll observations compare with corresponding in situ observations for v1. Note that the $r^{2}$ values are typically not high, since the ranges in chlorophyll values are low for each class. The figure highlights areas where priorities should be assigned for acquiring new data, according to high values of RMSD and bias and low number of observations. For example, we see that the optical water class 8 was characterised on the basis of only a handful of observations (in the case of v1) with low membership. We also see that the bias is highest in the most oligotrophic water class 1 and decreases towards the water classes with higher chlorophyll concentrations until it increases again for classes 6 and 7, which are more coastal in nature. On the other hand, RMSD is highest for optical class 6. The maps of dominant water classes can be used to identify geographic areas and seasons where the different optical classes are likely to be found, and such areas can be targeted for intensive field campaigns to collect more information and to reduce uncertainties. These initial results from v1 were used to improve subsequent versions by adding new observations. For example, class 8, which only had a handful of observations in v1, was characterised by over 100 observations in v4.

In the computation of uncertainties, we do not assume that the in situ observations are error-free. In fact, one might even argue that, at the scale of a satellite pixel, the satellite observation is the truth and that the real challenge is to devise in situ methods that would faithfully replicate the satellite observations at the appropriate time and space scales. However, regardless of the validity of such a position, it would side-step the true object of the work, which is to record the differences that a user might anticipate between in situ and satellite observations. We have, therefore, referred to these differences as uncertainties, rather than as errors.

## 12. Product Generation

A processing chain was then established to generate a time series of ocean-colour data for climate studies, using SeaWiFS, MODIS-Aqua, and MERIS data in the first instance, with VIIRS data added subsequently.

**Figure 5:**
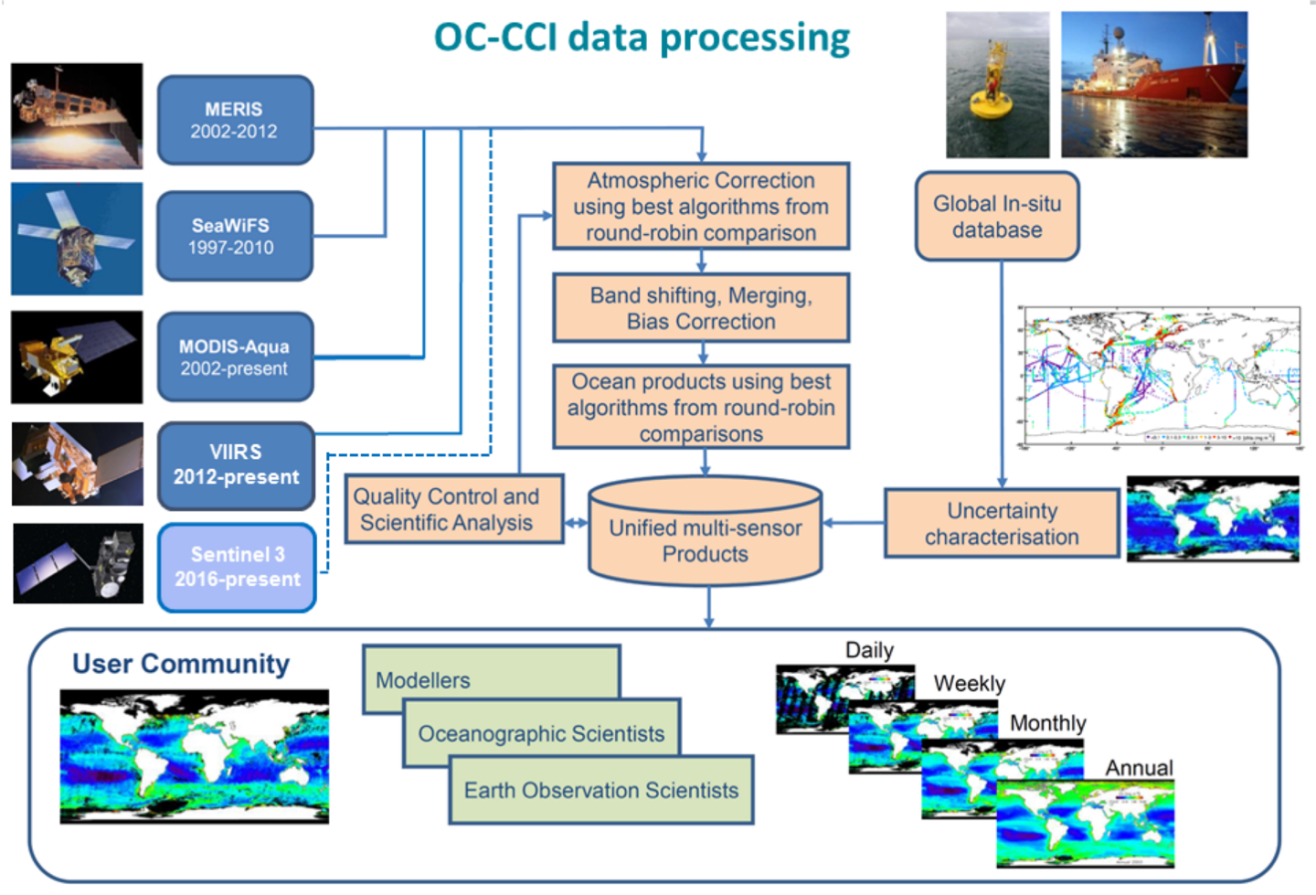
Schematic diagram illustrating the main steps in the processing chain implemented for OC-CCI: Note that VIIRS data were added to the input data stream from v3 onwards. Inclusion of OLCI is planned after quality evaluation, which is currently ongoing.

Based on user requirements [20], it was decided to produce daily products at 4 km resolution. For pragmatic reasons, the processing for different sensors started from different stages: for MODIS-Aqua, when L2Gen was selected for the processing chain (see Table 2 for a list of processors used in different versions of the processing chain), we used data already processed by NASA to level 3 binned, since they were available at the required 4 km resolution; SeaWiFS (GAC and LAC) data were processed from level 2 as they were not available at the 4 km level 3-binned stage; MERIS reduced resolution data (ESA 3rd reprocessing) were processed from level 1B with POLYMER to level 2 and then binned with the BEAM (http://www.brockmann-consult.de/cms/web/beam/) or SNAP (Sentinels Application Platform, https://www.brockmann-consult.de/portfolio/earth-scientific-image-processing/) software. Thus, the processing chain used here is not uniform with respect to the sensors—they joined the chain at different processing stages. From the level 3-binned stage onwards, all processing is unified. Cloud masks were applied to POLYMER level 2 outputs at the binning stage, and a database of in situ measurements was used to generate uncertainty tables. The main steps in the processing chain are illustrated in Figure 5.

A major challenge for generating the products was that the components are written in a range of languages and with different characteristics and constraints as they came from many sources and financial and time restraints precluded recoding. The interface of these different components clearly relied primarily on file interfaces, and an overall control framework was designed to orchestrate independent processes robustly through a chain of execution, identifying errors and ensuring they were not propagated. This approach [71] supports rapid prototyping and reprocessing, now a recognised requirement for all CCIs, and allowed (for example) changes to the bias correction scheme to be tested quickly. Although the approach accelerates integration and avoids the need to recode and re-validate existing scientific implementations, it does have efficiency costs. For OC-CCI, this could be overcome by the processing hardware, but future versions with, say, global 1 km resolution, may require a more memory- and CPU-efficient implementation.

The NetCDF (Network Common Data Form) outputs contain all products and use the NetCDF-4 classic model format, allowing transparent internal compression (critical for products of this volume), and comply with ESA CCI, CF (Climate and Forecast), and Unidata metadata conventions. In addition to the daily NetCDF products, 8-day and monthly composites and specific subsets of products are provided for all versions, as are pre-rendered images. A five-day composite was added from v3.1 onwards. The variables contained are chlorophyll-a concentration; normalised remote-sensing reflectance ($R_{rs}$ at 412, 443, 490, 510, 555, and 670 nm); vertical attenuation coefficient for downwelling irradiance $K_{d}\left(490\right)$; inherent optical properties ($a_{ph}$, $a_{dg}$, $b_{bp}$) at 412, 443, 490, 510, 555, and 670 nm; $a_{t}$, the total absorption coefficient, which is included for convenience, though it can be computed from the other components presented and the absorption by pure water itself; optical water classification (8 classes as in [64] with an additional class for coccolithophores, which represents the coccolithophore classes identified by [65] in v1, but expanded to 14 classes in v2 onwards, see [66]); and the number of observations (total and per sensor) at each pixel.

All products have associated per-pixel uncertainties (RMSD and bias), except for $a_{t}$ (convenience product), $b_{bp}$ (insufficient in situ data to make meaningful uncertainty estimates), and the water classes (not applicable).

The OC-CCI products are amongst the longest contiguous ocean-colour datasets produced to date. The scaling and robustness issues were addressed by splitting the problem at the natural breakpoint of single days, each encapsulated with all necessary data and logs. In the event of a problem, other containers were unaffected and all relevant debugging information is readily available. This structure lends itself naturally to batch processing on a cluster of commodity nodes. The total processing time is on the order of 2 months with approximately 100 nodes. The primary limitation is I/O demand due in part to the huge data volumes residing on large central file servers and being sent to networked clients for local processing. The use of Calvalus [71] on Apache Hadoop using Map-reduce and HDFS (Hadoop Distributed File System) technologies, which spread data across a cluster and move the processing to the data, helped overcome some of these problems with later versions of OC-CCI products.

## 13. Validation of Products

The RMSD and bias at the global scale for all paired observations are provided in Table 4 for the products (chlorophyll and $R_{rs}$). The results of the product validation have been described in detail in the Uncertainty Characterisation Document (UCD, OC-CCI, 2014c) and in the Product Validation and Intercomparison Report (PVIR, OC-CCI, 2014b).

## 14. Novel Features of the OC-CCI Time Series

### 14.1. Improved Coverage

An important characteristic of the OC-CCI products is the improved temporal and spatial coverage through the data processing approaches adopted, which include the merging of data from multiple missions and the use POLYMER for the atmospheric correction of one or more sensors; POLYMER is capable of retrieving data under sun-glint conditions, which substantially increases the spatial coverage of the data retrieved from MERIS. However, POLYMER was deemed suitable for processing only a subset of the sensors in each the versions thus far, and so, data gaps due to glint did occur for those sensors which were not processed with POLYMER. It is planned to generate an all-POLYMER version in the future on an experimental basis.

**Figure 6:**
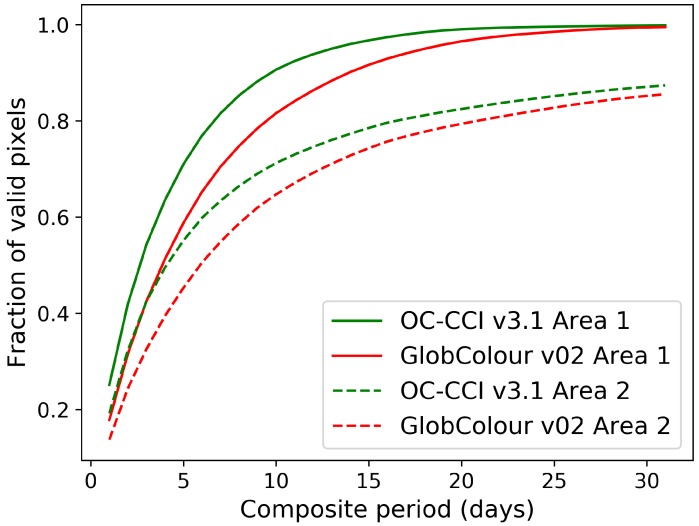
Number of days used to create a composite of $R_{rs}\left(443\right)$ and the corresponding fraction of valid pixels in the composite for two sites in the Atlantic: The average for the 2003–2010 period. Area one (continuous lines): one-degree box centred on 41${}^{\circ }$ N and 40${}^{\circ }$ W; area 2 (dashed lines): 11${}^{\circ }$ S and 4${}^{\circ }$ E. Green lines: OC-CCI-v3.1 product; red lines: GlobColour product. Consistency, traceability, and transparency are paramount OC-CCI requirements and, hence, in-water products are only calculated for those pixels with valid $R_{rs}$ data. It therefore follows that improved coverage in $R_{rs}$ is essential to ensure corresponding coverage in all in-water products.

The improved coverage has implications for studying phytoplankton dynamics under climate variability and change. For example, phytoplankton phenology can be studied at shorter time scales when coverage is better. As an example, Figure 6 shows the number of days used to create composites for one-degree boxes at two locations in the Atlantic against the fraction of pixels in the composite that had valid data, on average, for OC-CCI-v3.1 $R_{rs}\left(443\right)$ data. For comparison, results from a similar analysis on another merged dataset, GlobColour v02 (http://www.globcolour.info/), are also shown. If we say, for example, that at least half of the pixels in the area should have data before the composite can be considered representative of the area, then higher coverage would help achieve this target with a smaller number of days.

**Figure 7:**
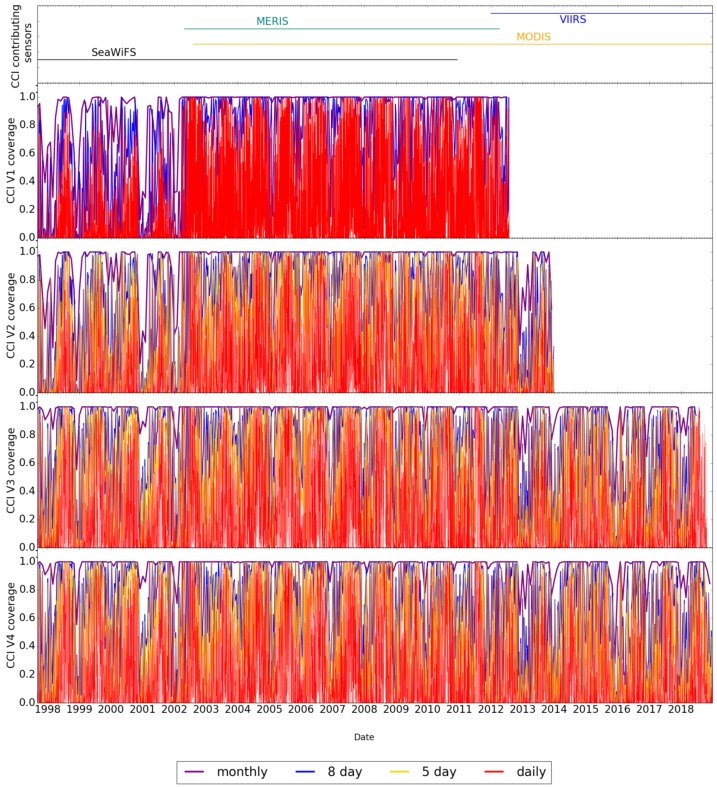
For the time series from September 1997 to December 2018, the number of pixels with valid chlorophyll data available from the various OC-CCI versions in a one-degree box centred on 41${}^{\circ }$N and 40${}^{\circ }$W, normalised to the number of pixels in the box, for 1-day, 5-day, 8-day, and 15-day composites: Five-day composites were not provided for v1. Note the improvement in coverage in SeaWiFS-only years in v3.1 and v4 compared with earlier versions because of incorporating SeaWiFS LAC data.

When only single sensors are contributing data (during the SeaWiFS-only period at the beginning of the time series and MODIS-Aqua-only period in v1), then the coverage is poor at times (Figure 7), even with 15-day composites. This highlights the importance of having two to three ocean-colour sensors in orbit at the same time if we are to meet the GCOS requirement for daily global data with adequate, representative coverage. We note that, in the context of climate studies, increased coverage should not be achieved at the cost of reduced accuracy because the datasets are designed, among other purposes, for studying small, long-term trends in ocean-colour products in a field where the climate signal may be masked by variability and oscillations in a broad spectrum of smaller time scales. A noisy and inaccurate product would make it even harder to identify trends.

**Figure 8:**
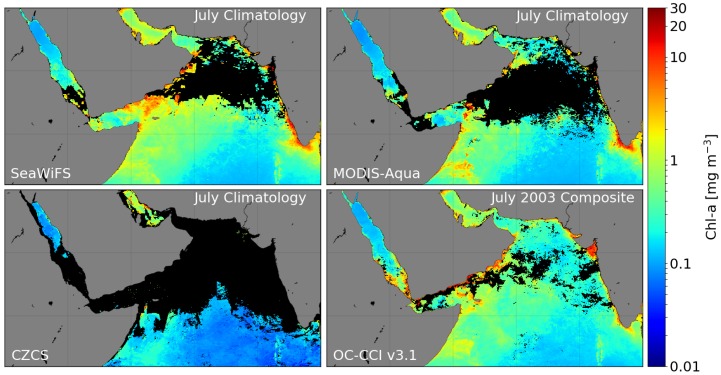
Monthly coverage of ocean-colour-derived chlorophyll data for the Arabian Sea. Black represents missing data. Top left panel: SeaWiFS July Climatology from NASA; top right panel: MODIS-Aqua July Climatology from NASA; bottom left panel: CZCS July Climatology from NASA; and bottom right panel: OC-CCI-v1.0 July monthly composite for one sample year (2003).

Similarly, Figure 8 shows that, for certain regions such as the Arabian Sea and the Red Sea, the spatial coverage in OC-CCI-v1.0 data is better compared with earlier single-sensor products because of the inclusion of MERIS data processed by POLYMER. In July, the coverage is particularly poor in the north-west part of the region in the chlorophyll climatologies for SeaWiFS, MODIS-Aqua, and the Coastal Zone Color Scanner (CZCS) from NASA archives compared with OC-CCI coverage. Note that the coverage shown for OC-CCI is for a particular year of the data and not the climatology. The coverage is, in fact, significantly better for all the MERIS years processed with POLYMER, making it possible to study inter-annual variations in the phytoplankton dynamics in the region during particular months that were previously sampled only poorly. Note that the comparison with MODIS-Aqua and SeaWiFS climatologies reveals that the improved coverage in OC-CCI products is achieved through incorporation of data processed with POLYMER. No doubt, the demise of MERIS and SeaWiFS (both well after their mission life expectancies) coupled with the delays in the follow-up missions have been detrimental to the OC-CCI project. However, the future looks promising, with the launch of the Sentinel 3A and B satellites carrying the OLCI (Ocean and Land Colour Imager) sensor in 2016 and 2018, respectively, which supplement the VIIRS family of sensors. The OLCI sensors are currently being evaluated for their incorporation into the OC-CCI time series.

### 14.2. Uncertainty Characterisation Based on Validation

OC-CCI products are the first of their kind, in that each of the products (except $b_{bp}$), generated from a merged time series of ocean-colour data, has uncertainties (bias and RMSD) assigned to every pixel based on validation of each of the products against corresponding in situ observations. A precursor to OC-CCI, GlobColour [72], has pixel-by-pixel uncertainties based on a theoretical computation that is available only for products derived from a bio-optical model. The advantages of the OC-CCI method are that the uncertainties are not based on geographic regions but on the optical type of water present; the basis of the assignment of uncertainties is the overall optical signal, which provides a common basis for all products; the total number of water types is small, so the number of observations per class would be greater than if we were to use a large number of class intervals; optical classification would eventually provide a basis for distinguishing between case-1 and case-2 waters (another user requirement); and the fuzzy logic avoids sharp boundaries across optical types. The disadvantages of the method is that sources of uncertainties such as high sun-zenith angle or high viewing angle or high aerosol optical thickness are not specifically considered through uncertainty characterisation, even though they are taken into account in the processing chain when flagging the data for quality. Another disadvantage is the limited in situ data available for each class, though this has been improving with each of the successive versions of OC-CCI products.

### 14.3. Merged Radiances that Band Shifted and Bias Corrected

A major goal of the OC-CCI project is to provide a multi-mission time series of remote-sensing reflectance ($R_{rs}$) data from which additional products such as the concentration of chlorophyll-a and inherent optical properties are derived. Furthermore, the OC-CCI project strives to fulfil the additional requirement that the merging process should minimise spurious temporal artefacts resulting from inter-mission differences. Indeed, climate research requires data records that are consistent in time, and therefore, an approach to correct for inter-mission differences (or biases) is needed. Existing ocean-colour missions have been measuring radiance values in different bands, which hinders a direct comparison of their respective $R_{rs}$ retrievals, for evaluating potential biases. For instance, the SeaWiFS, MERIS, and MODIS-Aqua centre wavelengths for the green band are 547, 555, and 560 nm, respectively. Addressing this problem imposed a shift in paradigm, from a focus on merging techniques to band-shifting and bias correction. A consequence of the approach is that a merged $R_{rs}$ data stream is produced first, to which a common set of algorithms is applied for retrieval of in-water properties.

The OC-CCI has implemented a band-shifting approach to correct for small differences in centre wavelengths of different sensors, which makes it possible to compare $R_{rs}$ from the various missions. The band-shifting approach relies on a two-step process, the calculation of inherent optical properties with the bio-optical algorithm selected by the OC-CCI, and the application of the same optical model in forward mode to compute $R_{rs}$ at the desired wavelength [53]. Expressing the $R_{rs}$ spectra on the same set of wavelengths (those of SeaWiFS) has allowed the first comprehensive inter-mission comparison for the full $R_{rs}$ spectrum. As the length of the time series grows, the problem of bias correction across nonoverlapping sensors could become more acute, especially if the sensor design and the set of wavebands available vary from sensor to sensor. The planned availability of two OLCI sensors in simultaneous orbit for many years into the foreseeable future should reduce the burden of the requirement for band-shifting.

The comparison has shown that inter-mission differences vary in space and time, and the largest part of inter-mission biases is accounted for by their spatial and seasonal dependence. Initially, OC-CCI data were corrected for the spatial dependence by removing a multi-annual (2003–2007) average bias for each $R_{rs}$ band and each grid point [73]. Subsequent versions incorporated a seasonal component to the correction. Albeit very simple (and progressively improved in OC-CCI-v2.0 and in OC-CCI-v3.1; see Table 2), the bias correction applied here effectively removed major components (spatial and seasonal) of the bias and reinforced the potential of the OC-CCI product set for time series analysis. Once band-shifting and bias correction have been implemented, merging is performed by simple averaging.

## 15. Concluding Remarks

In this paper, we have presented a series of activities undertaken to generate climate-quality ocean-colour products and to evaluate their quality and suitability for climate studies. We have summarised the results of the product validation against in situ observations. The key attributes of the products presented here that set them apart from precursor data include inter-sensor bias-corrected time series data, improved spatial and temporal coverage while retaining rigorous standards for data quality, and per-pixel uncertainty characterisation based on validation.

As intended, these products are finding broader applications. They are now part of the reprocessed product of Copernicus Marine Environmental Monitoring Services (CMEMS), with the services adding regionally tuned products for selected European waters to the global products from OC-CCI. A near-real-time analogue of OC-CCI products (not climate quality) is also available through CMEMS. An operational version of OC-CCI is also available now through the Copernicus Climate Change Services. The products described here were designed in consultation with marine ecosystem modellers, who are currently a primary user group for OC-CCI products, as seen, for example in Ciavatta et al. [74] and Dutkiewicz et al. [75] and in many more publications listed on the OC-CCI website.

But this is not the end; it is only the beginning. As algorithms improve, as novel products are developed to meet user requirements, and as input data streams are reprocessed by space agencies, the products presented here have to be re-evaluated and regenerated to reflect the state of the art. Thus, by May 2019, the OC-CCI data stream has already passed through four versions (Table 2). Additional work is needed to improve consistency in algorithms across sensors and to improve the performance of the algorithms in ultra-oligotrophic and eutrophic waters and in optically-complex coastal waters: according to the uncertainty characterisation by optical classes, these are the areas that need further attention. Updates are being made progressively to the optical water classification and the in situ database such that the uncertainty estimates are also being improved (Table 2). Incorporation of data from additional sensors has to be considered, to ensure continuity, and to improve spatial coverage, and notable here is the incorporation of VIIRS data in OC-CCI-v3.1 and the planned inclusion of data from Sentinel-3: the work presented here has highlighted the difficulties of achieving consistency across disparate sensors and the importance of having a consistent, well-calibrated series of ocean-colour satellites with MERIS-like spectral definition, functioning in operational mode. Sentinel-3 marks the beginning of such a time series. The data also show that a single sensor (SeaWiFS at the beginning of the time series) is insufficient to meet the user requirements for full global coverage on a daily basis. Better coverage requires at least two, or ideally, three sensors in orbit at the same time. In this regard as well, having two Sentinel-3 satellites working in constellation mode is commendable.

For generating a climate-quality time series, SeaWiFS provided the starting point. It was, in many respects, the best-understood sensor (because of the large number of studies, both evaluation-type and application-type that are available, based on SeaWiFS) when the OC-CCI work started. Hence, it was selected as the reference initially. Going forward, it would be important to shift the reference to OLCI spectral bands, to enable full exploitation of the additional capabilities provided by the sensor, and to take full advantage of the promise of an uninterrupted series of two sensors in orbit at any given time for the next couple of decades at least. However, this cannot be done until the two OLCI sensors in orbit now, on Sentinel-3A and Sentinel-3B, have been evaluated for climate quality. This work is currently ongoing. We envisage that, when the evaluation is completed, there will be a new OC-CCI time series that is anchored by OLCI. However, going backwards in time from the OLCI era, it is important not to generate data where none exist. Therefore, at present, it is envisaged that the $R_{rs}$ time series going backwards to SeaWiFS will only have the six SeaWiFS-like bands, though the MERIS years could, in principle, include all the MERIS bands that are compatible with OLCI; the challenge in this case is the bias correction between OLCI and MERIS, given that there is no temporal overlap between the two sensors in orbit.

Many contributors from outside the project have been key to the achieving the results presented here. A notable partner has been NASA and scientists engaged in NASA projects, who have given their time and help unstintingly. The broader user community, responding to requests for information, has helped shape the project. As we move into the next phase of the project, it is important to maintain user consultation and to continue to develop the products to meet the user requirements better. The problem is sufficiently complex that it would be foolhardy to imagine that any single group could solve all the problems and, hence, the importance of engaging and consulting international expert groups, notably the International Ocean-Colour Coordinating Group (IOCCG).

As envisaged from the start, the update process goes from user consultation to algorithm selection and then prototype development followed by product generation and validation, encapsulating a continual development-production-feedback loop and enabling continued science- and user-driven improvement in a short timeframe. This approach was invaluable for identifying issues rapidly, in integrating software and improving the outputs, and it is important to operate in this mode into the indefinite future. 

## Figures and Tables

**Table 1:** List of acronyms and notations used and their expansions and definitions.

Acronyms & Notations	Expansions & Definitions
δ	bias correction
λ	wavelength
μ	median ratio
$a_{dg}$	absorption coefficient of detrital particles and coloured dissolved organic matter (or gelbstoff) combined
$a_{ph}$	absorption coefficient of phytoplankton
$a_{t}$	total absorption coefficient
$b_{bp}$	back-scattering coefficient for particles
$K_{d}$	vertical attenuation coefficient for downwelling irradiance
$R_{rs}$	normalised remote-sensing reflectance
AOP	Apparent Optical Property
CCI	Climate Change Initiative
CF	Climate and Forecast
ECV	Essential Climate Variable
ESA	European Space Agency
GAC	Global Area Coverage
GCOS	Global Climate Observing System
HDFS	Hadoop Distributed File System
HPLC	High-Performance Liquid Chromatography
IOCCG	International Ocean Colour Coordinating Group
IOP	Inherent Optical Property
LAC	Local Area Coverage
L2Gen	NASA’s Level 2 Generator
MERIS	MEdium spectral Resolution Imaging Spectrometer
MERMAID	MERis MAtchup In Situ Database
MODIS-Aqua	Moderate-resolution Imaging Spectroradiometer-Aqua
NASA	National Aeronautics and Space Administration
NCEP	National Center for Environmental Prediction
NetCDF	Network Common Data Form
NOMAD	NASA bio-Optical Marine Algorithm Dataset
OC-CCI	Ocean-Colour Climate Change Initiative
OBPG	Ocean Biology Processing Group (of NASA)
PAR	Photosynthetically Available Radiation
POLYMER	POLYnomial based algorithm applied to MERIS
QAA	Quasi-Analytical Algorithm (QAA [19])
SVC	System Vicarious Calibration
SWIR	Short-wave infrared
VIIRS	Visible and Infrared Imaging Radiometer Suite

**Table 2:** Properties and modifications of processing steps for successive versions of Ocean-Colour Climate Change Initiative (OC-CCI): For further information on the OC-CCI Project and related documents, please visit esa-oceancolour-cci.org. Tools for data subsetting, visualisation, and downloading (ftp, web GIS, OPeNDAP, and composite browser) are also available at oceancolour.org (Supplementary Materials). Support is available from help@esa-oceancolour-cci.org. Access to other essential climate variable (ECV) data and a toolbox for data analyses are available through the climate change initiative (CCI) open data portal cci.esa.int/data.

Processing Step	Version 1	Version 2	Version 3.1	Version 4
Inputs	SeaWiFS GAC, MERIS, MODIS-A	SeaWiFS GAC, MERIS, MODIS-A	SeaWiFS GAC+LAC, MERIS, MODIS-A, VIIRS	SeaWiFS GAC+LAC, MERIS, MODIS-A, VIIRS
Input datasets	SeaWiFS: R2010.0; MODIS-A: R2013.1; MERIS: R3	SeaWiFS: R2010.0; MODIS-A: R2013.1; MERIS: R3	SeaWiFS: R2010.0; MODIS-A: R2014.0.1; MERIS: R3	SeaWiFS: R2018; MODIS-A: R2018; VIIRS: R2018; MERIS: R3
Atmospheric correction	POLYMER v2.7.0: MERIS; L2Gen 7.0: SeaWiFS, MODIS-A	POLYMER v3.0: MERIS;L2Gen: SeaWiFS, MODIS-A	POLYMER v3.5: MERIS, MODIS-A; L2Gen 7.3: SeaWiFS, VIIRS	POLYMER v4.8: MERIS;L2Gen v7.5: SeaWiFS, MODIS-A, VIIRS
in situ database	Initial version	Extended version with substantial increase in number of match-ups [22]	Further expanded in situ database	Further expanded in situ database [23]
Binning	Beam Binner: MERIS;L2Gen Binner: SeaWiFS, MODIS-A	Beam Binner v5 for all sensors, improving consistency; better binning algorithm	Further improvements in the binning algorithm to eliminate speckle	No change in binner from v3.1
Bias correction	Static correction per pixel	Incorporates improved seasonal variation in bias	Incorporates weekly composites, giving smoother, fuller correction	No change in bias correction from v3.1
Pixel identification	Idepix initial version: MERIS;L2Gen: SeaWiFS, MODIS-A	Idepix 2.0: SeaWiFS, MERIS;L2Gen: MODIS-A	Combination of Idepix and L2Gen	Combination of Idepix and L2Gen
Generation of optical classes	Used in situ $R_{rs}$ database	Used OC-CCI v2 data	Used OC-CCI v3.1 data	Used OC-CCI v4 data
in situ algorithms	Best performing algorithms selected globally	Best performing algorithms selected globally	Best performing algorithms selected for each optical class	Best performing algorithms selected for each optical class
Uncertainty characterisation	Used v1 classes and initial in situ database	Used v2 classes and improved in situ v2 database	Used v3.1 classes and improved in situ database	Used v4 classes and improved in situ v4 database
Quality assurance	Initial version, less automated	More automated quality assurance process	More automated quality assurance process	More automated quality assurance process
Length of time series	September 1997 to December 2012	September 1997 to December 2014	September 1997 to December 2015 (extended to December 2018)	September 1997 to December 2018
Doi:	10.5285/E32FEB53-5DB1-44BC-8A09-A6275BA99407	10.5285/b0d6b9c5-14ba-499f-87c9-66416cd9a1dc	10.5285/9c334fbe6d424a708cf3c4cf0c6a53f5	10.5285/00b5fc99f9384782976a4453b0148f49
How to cite the data	Sathyendranath et al. 2016 [24]	Sathyendranath et al. 2016 [25]	Sathyendranath et al. 2018 [26]	Sathyendranath et al. 2019 [27]

**Table 3:** Confusion matrix for pixel identification for v1.

		PixBox Data			
		Water	Cloud	Snow/Ice	Σ
**IdePix data**	water	5433	23	2	5458
	cloud	1033	15,068	2746	18,847
	snow/ice	2	66	1124	1192
	Σ	6468	15,157	3872	25,497

**Table 4:** Global uncertainty characteristics for oceanic properties derived from OC-CCI merged $R_{rs}$ data for v1 to v4: The results are based on comparison with matched in situ data. Number of match-up observations *N* that have been used for each of the properties is given. Note that, for chlorophyll-a (Chl-a, in mg m${}^{-3}$), the analyses are done on log_10_-transformed data. The units of $R_{rs}$ are sr${}^{-1}$.

Variable		v1	v2	v3.1	v4
log${}_{10}$(Chl-a)	RMSD	0.303	0.328	0.314	0.340
	Bias	−0.0191	−0.0284	−0.00662	−0.0409
	$r^{2}$	0.81	0.79	0.76	0.73
	*N*	6049	7958	14,582	18,055
$R_{rs}\left(412\right)$	RMSD	0.00128	0.00138	0.00130	0.00130
	Bias	8.68 $\times \;10^{-5}$	2.60 $\times \;10^{-4}$	3.94 $\times \;10^{-4}$	−7.44 $\times \;10^{-5}$
	$r^{2}$	0.87	0.87	0.89	0.88
	*N*	14,485	16,594	17,249	29,964
$R_{rs}\left(443\right)$	RMSD	0.00114	0.00113	9.12 $\times \;10^{-4}$	0.00111
	Bias	−1.19 $\times \;10^{-5}$	−1.35 $\times \;10^{-5}$	8.21 $\times \;10^{-5}$	−9.36 $\times \;10^{-5}$
	$r^{2}$	0.83	0.81	0.86	0.83
	*N*	12,711	19,128	18,614	32,186
$R_{rs}\left(490\right)$	RMSD	0.00125	0.00101	0.00111	0.00106
	Bias	3.59 $\times \;10^{-4}$	2.91 $\times \;10^{-4}$	4.60 $\times \;10^{-4}$	2.61 $\times \;10^{-4}$
	$r^{2}$	0.76	0.77	0.79	0.79
	*N*	15,112	21,346	21,794	34,546
$R_{rs}\left(510\right)$	RMSD	0.000934	0.000658	0.000557 $\times \;10^{-4}$	0.000678 $\times \;10^{-4}$
	Bias	1.12 $\times \;10^{-4}$	2.45 $\times \;10^{-4}$	2.49 $\times \;10^{-4}$	2.24 $\times \;10^{-4}$
	$r^{2}$	0.54	0.45	0.36	0.47
	*N*	3272	14,100	13,332	17,441
$R_{rs}\left(555\right)$	RMSD	0.00155	0.00107	0.00132	0.00105
	Bias	6.23 $\times \;10^{-4}$	3.04 $\times \;10^{-4}$	5.30 $\times \;10^{-4}$	2.73 $\times \;10^{-4}$
	$r^{2}$	0.76	0.84	0.87	0.85
	*N*	7490	14,862	15,194	17,557
$R_{rs}\left(670\right)$	RMSD	0.000556	0.000401	0.000435	0.000473
	Bias	−2.49 $\times \;10^{-5}$	7.62 $\times \;10^{-5}$	1.43 $\times \;10^{-4}$	1.11 $\times \;10^{-4}$
	$r^{2}$	0.68	0.77	0.80	0.78
	*N*	5950	9429	9764	18,439

## Supplementary Materials

OC-CCI data are available from: https://www.oceancolour.org/.
